# Detection of the 40 Hz auditory steady-state response with optically pumped magnetometers

**DOI:** 10.1038/s41598-022-21870-5

**Published:** 2022-10-26

**Authors:** Kyung-min An, Jeong Hyun Shim, Hyukchan Kwon, Yong-Ho Lee, Kwon-Kyu Yu, Moonyoung Kwon, Woo Young Chun, Tetsu Hirosawa, Chiaki Hasegawa, Sumie Iwasaki, Mitsuru Kikuchi, Kiwoong Kim

**Affiliations:** 1 Research Center for Child Mental Development, Kanazawa University, Kanazawa, 920-8640 Japan; 2 Division of Socio-Cognitive-Neuroscience, Department of Child Development, United Graduate School of Child Development, Osaka University, Kanazawa University, Hamamatsu University School of Medicine, Chiba University and University of Fukui, Kanazawa, 920-8640 Japan; 3 Korean Research Institute of Standards and Science, Daejeon, 34113 Republic of Korea; 4 Centre for Human Brain Health, School of Psychology, University of Birmingham, Birmingham, B15 2TT UK; 5 Department of Psychology, Chungnam National University, Daejeon, 34134 Republic of Korea; 6 Department of Physics, Chungbuk National University, 1 Chumgdae-ro, Seowon-gu, Cheongju, Chungbuk 28644 Republic of Korea

**Keywords:** Neuroscience, Auditory system

## Abstract

Magnetoencephalography (MEG) is a functional neuroimaging technique that noninvasively detects the brain magnetic field from neuronal activations. Conventional MEG measures brain signals using superconducting quantum interference devices (SQUIDs). SQUID-MEG requires a cryogenic environment involving a bulky non-magnetic Dewar flask and the consumption of liquid helium, which restricts the variability of the sensor array and the gap between the cortical sources and sensors. Recently, miniature optically pumped magnetometers (OPMs) have been developed and commercialized. OPMs do not require cryogenic cooling and can be placed within millimeters from the scalp. In the present study, we arranged six OPM sensors on the temporal area to detect auditory-related brain responses in a two-layer magnetically shielded room. We presented the auditory stimuli of 1 kHz pure-tone bursts with 200 ms duration and obtained the M50 and M100 components of auditory-evoked fields. We delivered the periodic stimuli with a 40 Hz repetition rate and observed the gamma-band power changes and inter-trial phase coherence of auditory steady-state responses at 40 Hz. We found that the OPM sensors have a performance comparable to that of conventional SQUID-MEG sensors, and our results suggest the feasibility of using OPM sensors for functional neuroimaging and brain–computer interface applications.

## Introduction

An auditory steady-state response (ASSR) is the result of the entrained neural rhythm in the primary auditory region generated by the periodic repetition of an auditory stimulus^1^. In humans, the ASSR is known to have a maximum magnitude at approximately 40 Hz, which is the resonance frequency of the auditory neural circuit^2–4^. The amplitude and phase of 40 Hz ASSR are supposed to reflect the balance between the inhibitory GABAergic and excitatory glutamatergic neurons^5,6^.

Two methods have been used to investigate 40 Hz ASSR, namely the event-related spectral perturbation (ERSP) and inter-trial phase coherence (ITPC) methods. The ERSP is a measure of induced power changes and is independent of the phase. The ITPC is a measure of the phase synchronization across trials and is also called the phase-locking factor^7,8^.

Reduced power and phase synchronization of the 40 Hz ASSR have been reported in individuals with schizophrenia^6,9^, bipolar disorders^10,11^, and autism spectrum disorders^12,13^.

The 40 Hz ASSR can be non-invasively measured through scalp electroencephalography (EEG) and magnetoencephalography (MEG). EEG and MEG measure neurophysiological activities with high temporal resolution. EEG has the advantages of a relatively simple and cost-effective system and the flexible arrangement of sensors. However, EEG has a longer preparation time for attaching electrodes on the scalp and has limited spatial resolution owing to the low and inhomogeneous electrical conductivity of the skull^14^. MEG has a high spatial resolution because the neuro-magnetic field is not sensitively affected as it passes through head tissue^15,16^. Conventional MEG measures magnetic fields generated by neurons using superconducting quantum interference devices (SQUIDs). Low-temperature SQUID sensors usually operate at approximately 7 K with the use of liquid helium. A rigid reservoir for the liquid helium is required to maintain a cryogenic temperature. The use of the MEG Dewar flask requires the SQUID sensors to be fixed inside a helmet, and the distance between the sensors and scalp is at least approximately 2 cm.

Recently, optically pumped magnetometers (OPMs) with a small size of 12.4 mm × 16.6 mm × 24.4 mm have been developed and commercialized^17,18^. The OPM sensor operates at room temperature and can be placed close to the scalp in a flexible manner. OPM sensors have been applied to detect neuromagnetic signals with such advantages. Previous OPM-based MEG studies have measured various brain activities relating to auditory-evoked fields (AEFs)^19–22^, visual processing^23^, somatosensory processing^24^, motor processing^25^, and language function^26,27^.

There is, however, no report of the OPM-MEG measurement of the 40 Hz ASSR, which reflects the functions of gamma-band activity and has potential clinical application. In this study, we developed an OPM-MEG system using six OPM sensors to detect auditory brain responses from the temporal lobe. We presented participants with auditory pure-tone bursts while conducting OPM-MEG recordings and confirmed that the OPM sensors can detect the AEFs. Additionally, we delivered repetitive auditory stimuli at 40 Hz and demonstrated that the OPM can reliably detect the 40 Hz ASSR by calculating the ERSP and ITPC.

## Materials and methods

### Participants

Twenty-two right-handed healthy participants (mean age: 27.05 ± 4.36 years; 11 females) participated in the study. Handedness was assessed using a translated version of the Edinburgh Handedness Inventory^28^. All participants had normal hearing and normal or corrected-to-normal vision and no participant reported any neurological or psychiatric disorder. The experimental procedures were approved by the Ethics Committee of the Korea Research Institute of Standards and Science (KRISS-IRB-2021-04). All participants gave their written informed consent. All experiments were performed in accordance with relevant guidelines and regulations.

### Experimental paradigm and stimuli

We presented two types of auditory stimulus during the OPM-MEG recordings. We first tested pure-tone auditory stimuli to confirm that OPM-MEG can detect the AEFs. The pure-tone stimulus was a 1 kHz tone burst with a duration of 100 ms. We delivered pure-tone bursts 230–240 times with an inter-stimulus interval of 1.8–2.3 s in one session.

We used auditory click-train sounds to elicit the ASSR at gamma frequency. The auditory click-train was created with 1-ms pulse sounds delivered at 40 Hz for 1 s. We presented a total of 250 click-train stimuli with an inter-stimulus interval of 2.5–3 s in two sessions. Each session lasted approximately 6 min.

During the recordings, participants stared at a fixation point and the auditory stimuli were presented at 80 dB to the right ear through a MEG-compatible ear tube. The stimuli were randomly delivered to avoid habituation, and we asked the participants to count the number of stimuli delivered in each session to help the participants to concentrate on the stimuli.

### OPM-MEG acquisition

**Figure 1:**
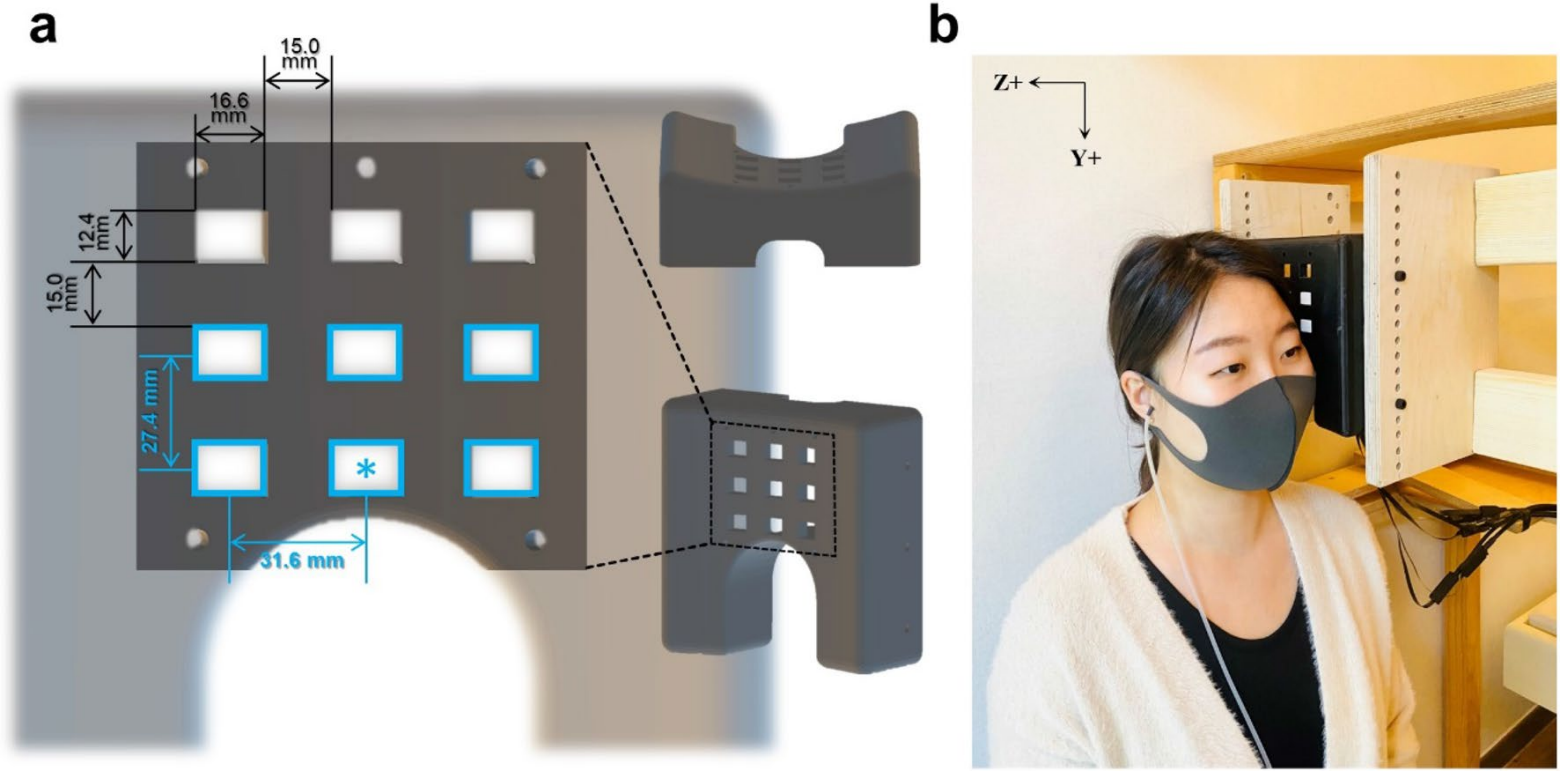
OPM sensor array and measurement setup in the magnetically shielded room. (**a**) We developed a sensor array for arranging six OPM sensors. The OPM sensor array had nine sockets in which to insert OPM sensors. Six OPM sensors were fixed in the sockets marked by blue rectangles. The center sensor (marked with an asterisk) was positioned on the participant’s T3 point, which was overlying the left auditory cortex according to the EEG sensor layout. (**b**) The OPM sensor array was positioned to cover the temporal region of the left side of the participant’s head while the participant was sitting in the magnetically shielded room. The auditory stimuli were delivered through an ear tube to the participant’s right ear.

OPM-MEG was measured using an array of six OPMs (Gen-2.0 QZFM; QuSpin Inc., Louisville, CO). The measurement setup is shown in Fig. 1. The OPM sensors were mounted on a three-dimensionally printed curved plate that fitted the temporal head surfaces of the participants. The plate had an arched hollow on the bottom side on which to place a participant’s left ear. This hollow made it comfortable for the participant to lean their head close to the sensor plate. The OPM sensor plate had nine sockets with separations of 15 mm in three rows and three columns. The six OPM sensors were fixed in the sockets of the two lower rows (indicated by blue rectangles in Fig. 1a). The center-to-center distance between adjacent OPM sensors was 31.6 mm horizontally and 27.4 mm vertically.

To find the appropriate sensor position for detecting the auditory brain signals, we marked the T3 and Cz points of each participant according to the EEG 10–20 lead system. The T3 point is the scalp site overlying the left-hemisphere auditory area of the cerebral cortex. The Cz point is the midline central point of the scalp. We placed the center sensor (indicated by the blue asterisk in Fig. 1a) on the T3 point of each participant and aligned the vertical axis of the sensor array along the line between T3 and Cz. The participants leaned toward the sensor array and headrest such that their head was close to the sensors (Fig. 1b). During the OPM-MEG recordings, the participants were seated in a two-layer magnetically shielded room (MSR) (Korea Research Institute of Standards and Science, Republic of Korea). We checked the empty-room noise in several MSRs and decided to conduct our experiment in a two-layer MSR underground due to its superior noise properties. The empty-room noise in the two-layer MSR was approximately 15–20 fT/√Hz in the 2–80 Hz frequency range. Empty-room data with unfiltered signals is shown in Supplementary Fig. 1. No reference sensors or compensation coils were used in the experimental setup.

The electronics controller of the OPM system delivered two analogue outputs for the magnetic field strength in the y- and z-directions for each sensor. The analog signals and auditory trigger were simultaneously sampled by a 16-bit data acquisition system (NI-9205, National Instruments Co., Austin, TX) at a sampling rate of 1 kHz. The scaling of the output voltage to the measured magnetic field was 2.8 V/nT. We used only the signals of the z-direction in our analysis.

### Data analysis

We analyzed the OPM-MEG data using the Brainstorm toolbox^29^, FieldTrip toolbox^30^, and MATLAB (The MathWorks). Raw data were bandpass filtered from 0.2 to 100 Hz. We applied a powerline notch filter at 60 Hz and band-stop filters at 21.5 and 27 Hz (± 0.5 Hz), which are the frequencies of environmental vibration noise. We segmented data from − 3 to 3 s following the onset of each auditory stimulus. We rejected the trials containing obvious artifacts over 300 fT.

To calculate the event-related fields, we applied a low-pass filter to the data of pure-tone bursts with a cut-off frequency of 40 Hz and to the data of the 40 Hz ASSR at 60 Hz. The individual AEFs were DC normalized with the baseline from − 200 to 0 ms according to the auditory stimulus onset. We averaged AEFs within each participant and across all participants to obtain the grand-average AEFs. To obtain the field distribution for the AEFs of pure-tone bursts, we calculated magnetic field maps of the baseline (− 110 to − 100 ms according to the auditory stimulus onset), M50 (40–50 ms), and M100 (80–90 ms) components.

To calculate the power changes and phase synchronization of the ASSR, we applied time–frequency analysis at 1–60 Hz using a seven-cycle Morlet wavelet for each trial. The time–frequency representations (TFRs) were calculated by converting to the percentage changes in power relative to the baseline (− 1.1 to − 0.1 s). TFRs were averaged for each participant and then grand-averaged for all participants. We assessed the significant time–frequency component related to the ASSR by comparing with the baseline period (− 1.1 to − 0.1 s) applying a parametric t-test (two-tailed). A correction of the false discovery rate was applied to control for type I error in the t-test. The alpha level was set at 0.05 in the statistical analysis. We calculated the gamma-band response modulated at 40 Hz by averaging the power changes from 38 to 42 Hz.

After decomposing the clean trial data using Morlet wavelets at 1–60 Hz, we calculated the ITPC as^7^

$$ITPC\left(f,t\right)=\frac{1}{n}\sum_{k=1}^{n}\frac{Fk\left(f,t\right)}{|Fk\left(f,t\right)|}$$

where *t* is time, *f* is the frequency, *n* is the number of trials, and *Fk*(*f*, *t*) is the spectral estimate of trial *k* at frequency *f* and time *t*. The ITPC reflects the phase synchronization at each time–frequency point. ITPC values range from 0 to 1 for a given frequency and time point. Larger ITPC values represent higher consistency in the phase synchronization and smaller ITPC values represent lower phase synchronization across trials^31^.

### Ethics approval and consent to participate

This study was approved by the Ethics Committee of the Korea Research Institute of Standards and Science. After receiving a complete explanation of the study, all participants provided full written informed consent.

## Results

**Figure 2:**
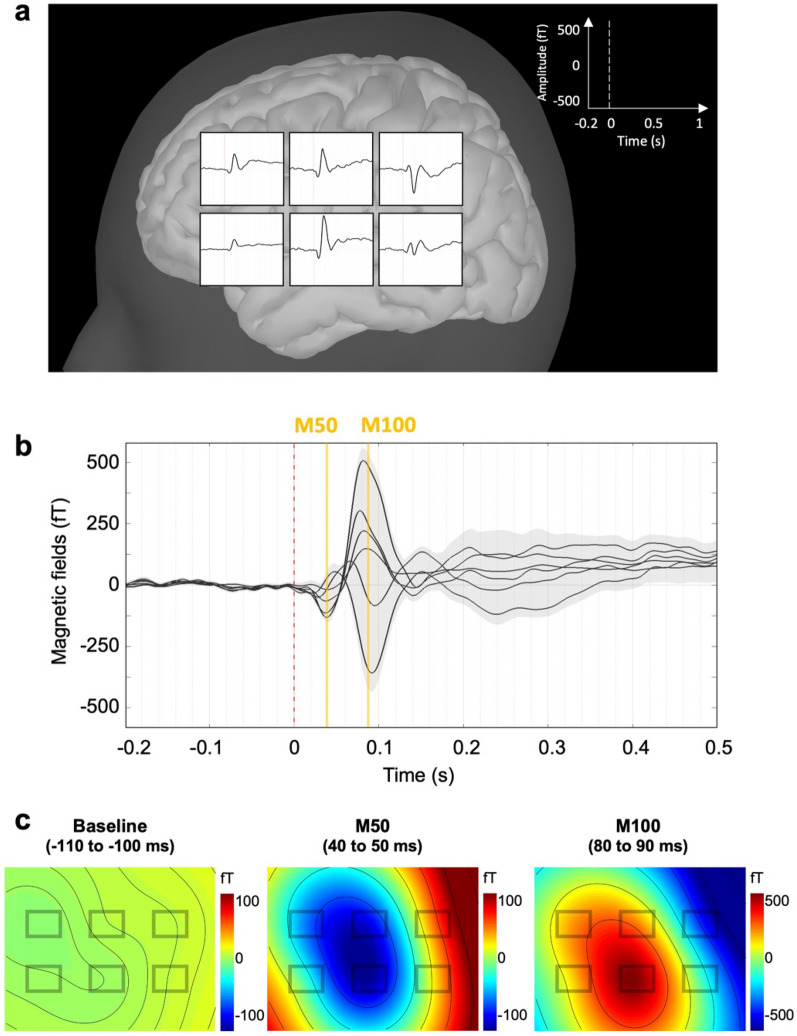
Auditory-evoked fields and their topographical distributions recorded by OPM sensors during auditory pure-tone bursts. (**a**) Grand-average auditory-evoked field of 22 participants measured by each OPM sensor. (**b**) M50 component observed at approximately 43 ms (42.91 ± 6.12 ms) and M100 component observed at approximately 86 ms (86.27 ± 7.34 ms). A light-grey area represents the standard error across all participants. (**c**) Topographical maps representing the field distributions of the baseline (− 110 to − 100 ms according to the onset of the auditory pure-tone burst), M50 component (40–50 ms), and M100 component (80–90 ms).

We first delivered pure-tone bursts and calculated AEFs to confirm that our OPM sensor array could detect brain auditory activities. We observed clear AEFs detected by the six OPM sensors. Figure 2a presents the sensor distributions of the grand-average AEFs across the 22 participants. We obtained maximum activities of AEFs from the center sensor, which covered the T3 scalp site. Figure 2b shows the clear M50 and M100 components. The M50 component appeared at approximately 43 ms (42.91 ± 6.12 ms) whereas the M100 component appeared at approximately 86 ms (86.27 ± 7.34 ms). Figure 2c presents the topological map patterns of the baseline period (− 110 to − 100 ms according to the auditory stimulus onset), M50 component (40–50 ms), and M100 component (80–90 ms). The topologies of the M50 and M100 components had opposing polarity. Supplementary Fig. 2 shows the individual AEFs of the representative participants.

We presented auditory click-train stimuli at 40 Hz to the participants to investigate the modulated auditory gamma-band activity. The duration of the auditory click-train stimuli was 1 s. We calculated event-related fields, TFRs, and ITPC to investigate the 40 Hz ASSR.

**Figure 3:**
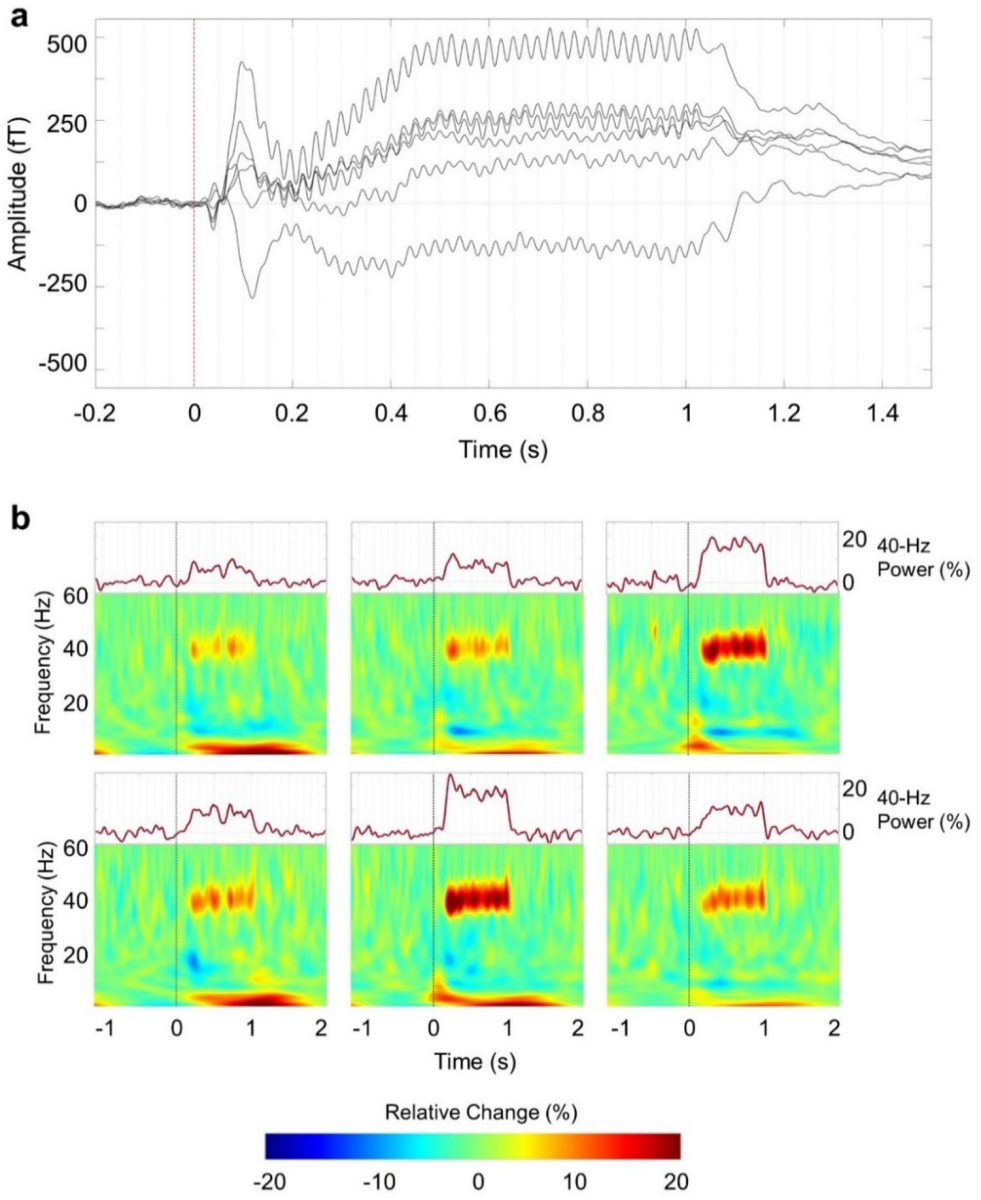
Grand-average waveforms and time–frequency representations during the 40 Hz auditory steady-state response. (**a**) Grand-average waveforms recorded by the six OPM sensors show that the magnetoencephalographic fields were modulated by the repetitive auditory stimuli at 40 Hz. (**b**) Grand-average time–frequency representations show that the relative power increased in the 40 Hz gamma band compared to baseline power. Upper panels show the mean power changes of the gamma frequency band at 38–42 Hz. The 40 Hz gamma power increased for 1 s when 40 Hz auditory steady-state response stimuli were presented.

Figure 3a shows the grand-average AEFs related to the repetitive auditory stimuli at 40 Hz. We observed the M50 and M100 components at the early response and found brain waveforms modulated at 40 Hz lasting for 1 s.

Group-averaged TFRs for the repetitive auditory stimuli at 40 Hz are plotted for each OPM sensor (lower panel of Fig. 3b). The power of the gamma-band response increased at 40 Hz for about 1 s. The upper panel of Fig. 3b shows the power changes of the 40 Hz ASSR obtained by averaging from 38 to 42 Hz. Supplementary Fig. 3 shows the individual power changes of the 40 Hz ASSR of the representative participants. We see that the gamma-band responses increased during the presentation of the 40 Hz auditory click-train stimuli (Supplementary Fig. 4).

**Figure 4:**
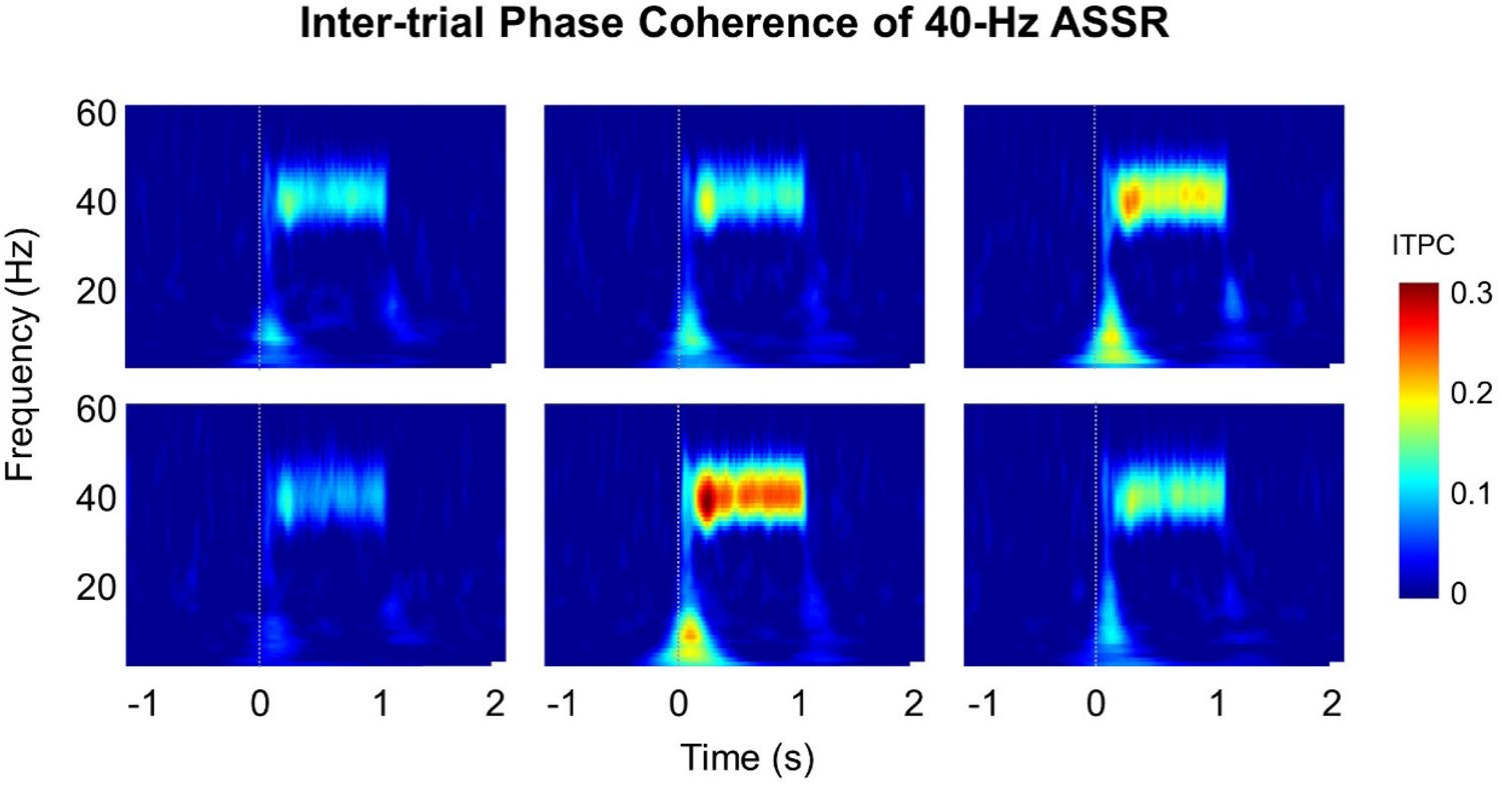
Results of inter-trial phase coherence of the 40 Hz ASSR. Inter-trial phase coherence maps of each sensor show the trial-to-trial phase-locking at 40 Hz.

We analyzed the ITPC to investigate the phase synchronization of the 40 Hz ASSR. Figure 4 shows the results of the ITPC for each OPM sensor. We find strong phase-locking at 40 Hz across trials. All our results show a maximum value for the center sensor, which overlaid the T3 point.

In the results, we provide grand-averaged results to show tendencies of the brain responses across all participants. In addition, we provide individual results from the representative participants in the Supplementary figures.

## Discussion

This was the first study to measure the ASSR using OPM sensors to the best of our knowledge. We recorded auditory brain activities using six OPM sensors inside of a two-layer magnetically shielded room. We observed the AEFs related to the pure-tone burst stimuli and the field distributions. We found obvious power changes and phase synchronization of the ASSR modulated at 40 Hz.

In this study, we measured the AEFs using OPM sensors and confirmed that our results replicate the findings of previous OPM studies^19–22^. We found clear M50 and M100 components of AEFs and obtained the topological map pattern. We further found that the M50 and M100 components had topologically opposite polarity. The brain source of the M50 component is known to be oriented toward the anterior/dorsal of the head whereas that of the M100 component is oriented toward the posterior/ventral of the head. These components were oriented approximately in opposite directions and had opposite topological patterns.

Our study showed the obvious power enhancement and phase synchronization of the ASSR at 40 Hz. These results are consistent with the findings of previous EEG and MEG studies^1,3,32^. The auditory cortex is known to have a resonance frequency of approximately 40 Hz in humans^2–4^. The 40 Hz ASSR is an evoked neural rhythm that is entrained by the external repeated auditory stimuli^1^. The atomic signal gain of an OPM intrinsically depends on the detection frequency^33^. Thus, the phase analysis of measured brain signals using an OPM is supposed to be questionable. However, as in the case of the 40 Hz ASSR described in this article, a narrow-band analysis provides a reasonable phase synchronization result and power change. This study thus provides the groundwork for further neuronal phase analysis in OPM-MEG studies.

In this study, we obtained clear brain activities even though we measured the OPM-MEG signals in a two-layer magnetically shielded room. These clear signals might result from the close separation of the OPM sensors and source of the brain signals. OPM sensors can be placed near the scalp with a gap of approximately 3 mm because the OPM sensor operates at room temperature. The strength of the magnetic field is approximately inversely proportional to the distance squared. OPM sensors therefore record clear signals owing to their proximity to the signal source.

We recorded auditory brain activities using relatively few OPM sensors. We placed six OPM sensors so as to individually cover the T3 point with the central sensor; the T3 point is the scalp site of EEG overlying the left-hemisphere auditory area of the cerebral cortex. We found that the auditory brain signals were strongest for the sensor over the T3 point. The OPM sensor is compact and has flexible placement. These features allow personalized sensor arrangements according to the head size and head shape, as seen for EEG sensors. The OPM sensor can be used to measure the neuromagnetic field in a personalized position according to the head size and shape with a small number of sensors. Its use would therefore minimize the burden on the participant during recording, especially for child participants. In the present study, we analyzed data at the sensor space according to the individual EEG sensor location, because we measured brain activity using a small number of OPM sensors. In future studies, it will be necessary to use more sensors to perform source localization by registration of the sensors to individual MRI.

In this study, we measured the AEFs and 40 Hz ASSR using OPM sensors in healthy participants. The latency and/or amplitude of AEFs has been related to child development^34–36^ and neurodevelopmental disorders^37–39^. The 40 Hz ASSR has shown high test–retest reliability^32,40^ and has been considered as a useful indicator for neurophysiological disorders, such as schizophrenia^6,9^, bipolar disorder^10,11^, and autism spectrum disorders^12,13^. The OPM sensor can be used in a flexibly fitting sensor array for small heads with only a small number of sensors over the region of interest of the brain area. Therefore, it is potentially an effective tool for child development and clinical research. We hope that our results will provide the groundwork for future OPM-MEG studies on child development, clinical practice, and brain–computer interfaces.

## Supplementary Information

Supplementary Information.

## Data Availability

The datasets generated and/or analyzed in the current study are not publicly available as they contain information that could compromise the privacy of research participants but are available from the corresponding author on reasonable request. The data supporting the findings of this study are available on request from the corresponding author, K.A. The data are not publicly available as they contain information that could compromise the privacy of the research participants.
