# Supplementary material for: Detection of the 40 Hz auditory steady-state response with optically pumped magnetometers
Source: Sci Rep. 2022 Oct 26;12:17993. doi: 10.1038/s41598-022-21870-5 (PMC9606299; doi:10.1038/s41598-022-21870-5)
Supplement: Supplementary file 1 — Supplementary Information. [file 41598_2022_21870_MOESM1_ESM.docx]

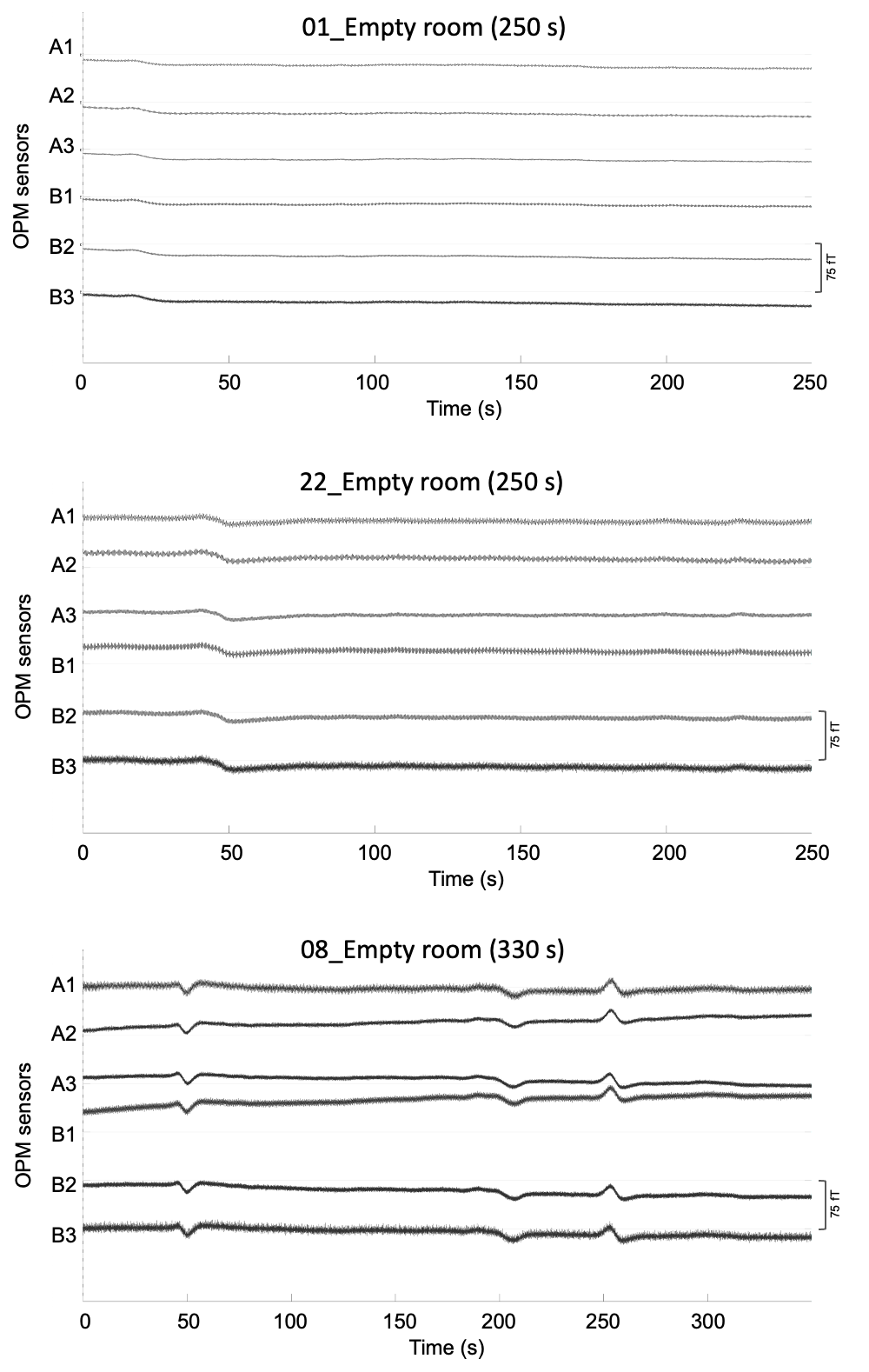


**Supplementary Figure 1. Empty-room data with unfiltered signals.** Although there were several fluctuations due to the strong external magnetic noise, the signals were generally stable.

**
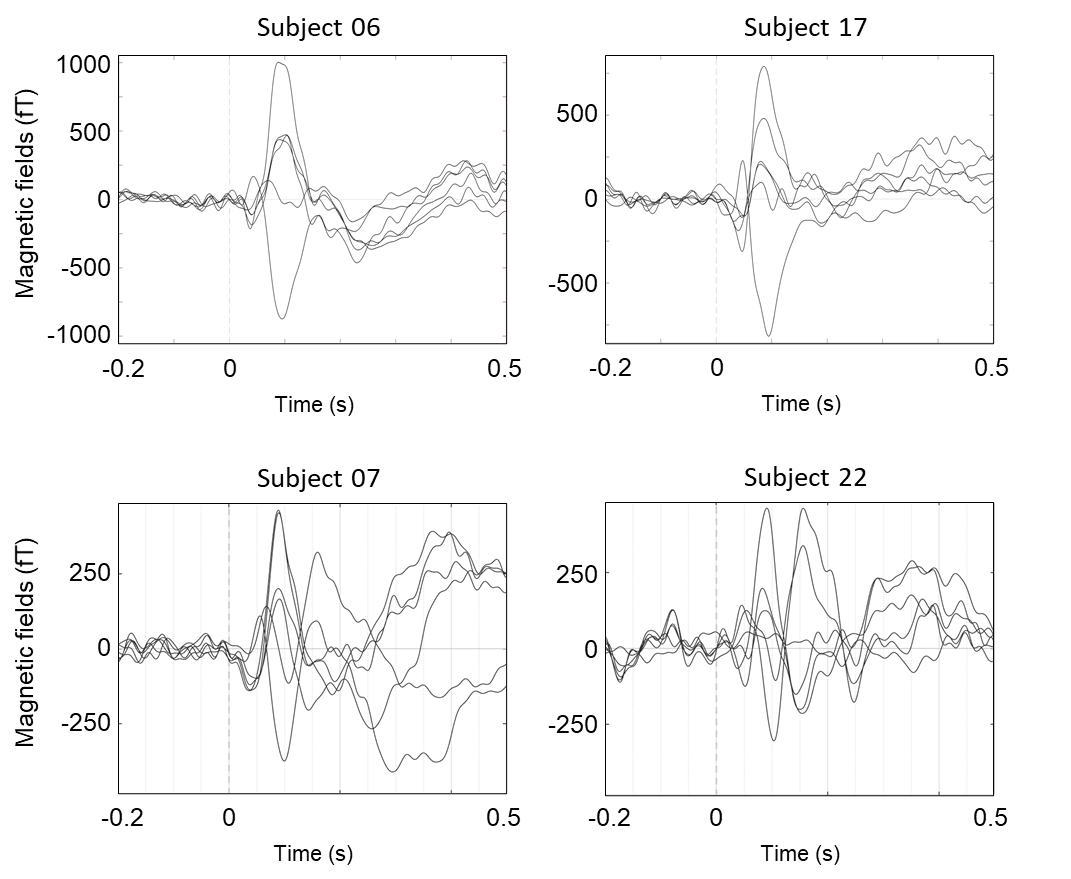
**

**Supplementary Figure 2. Auditory-evoked fields related to the pure-tone bursts in the representative participants.**

**
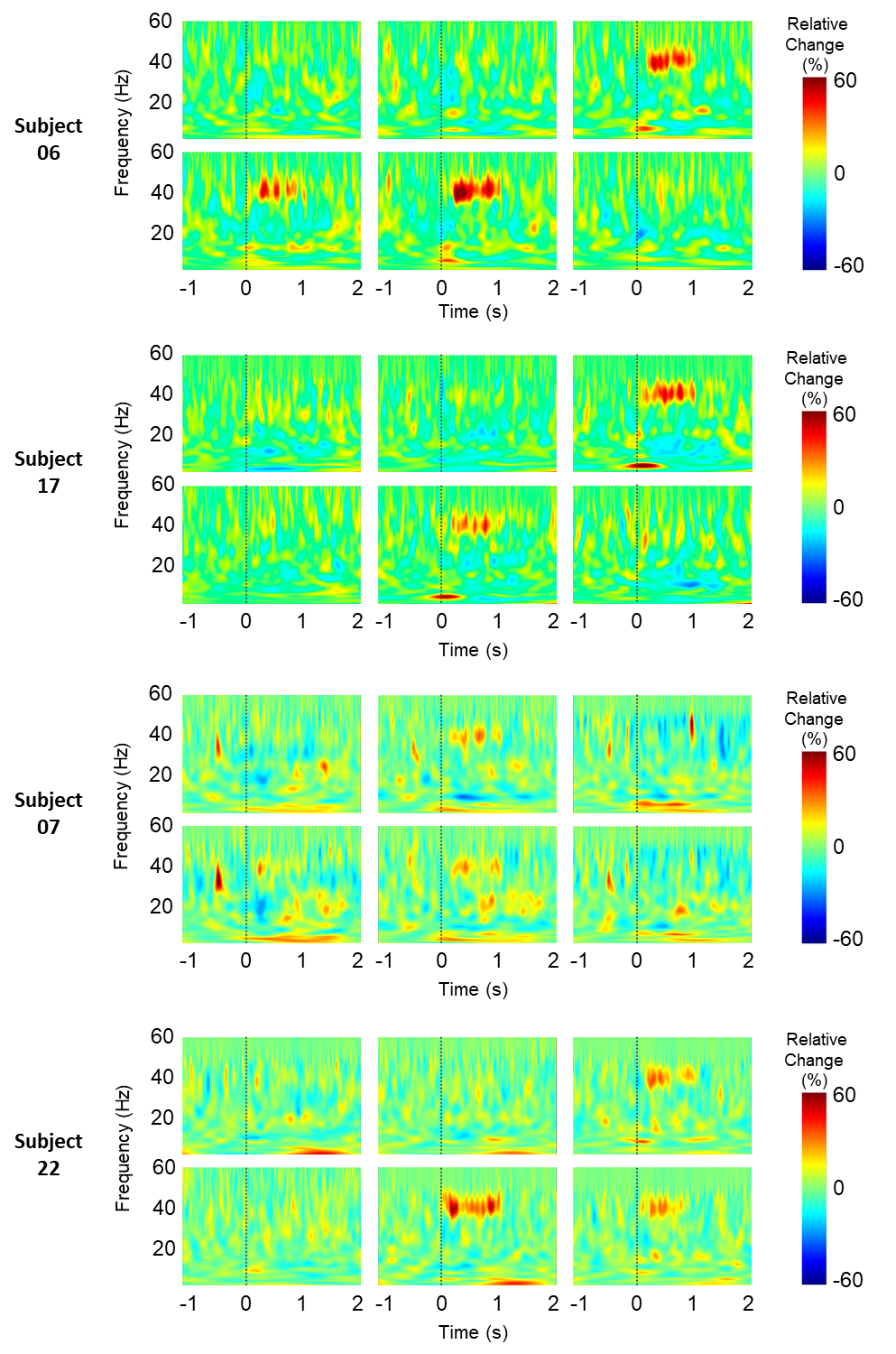
**

**Supplementary Figure 3. Time–frequency representations during the 40 Hz auditory steady-state response in the representative participants.**

**
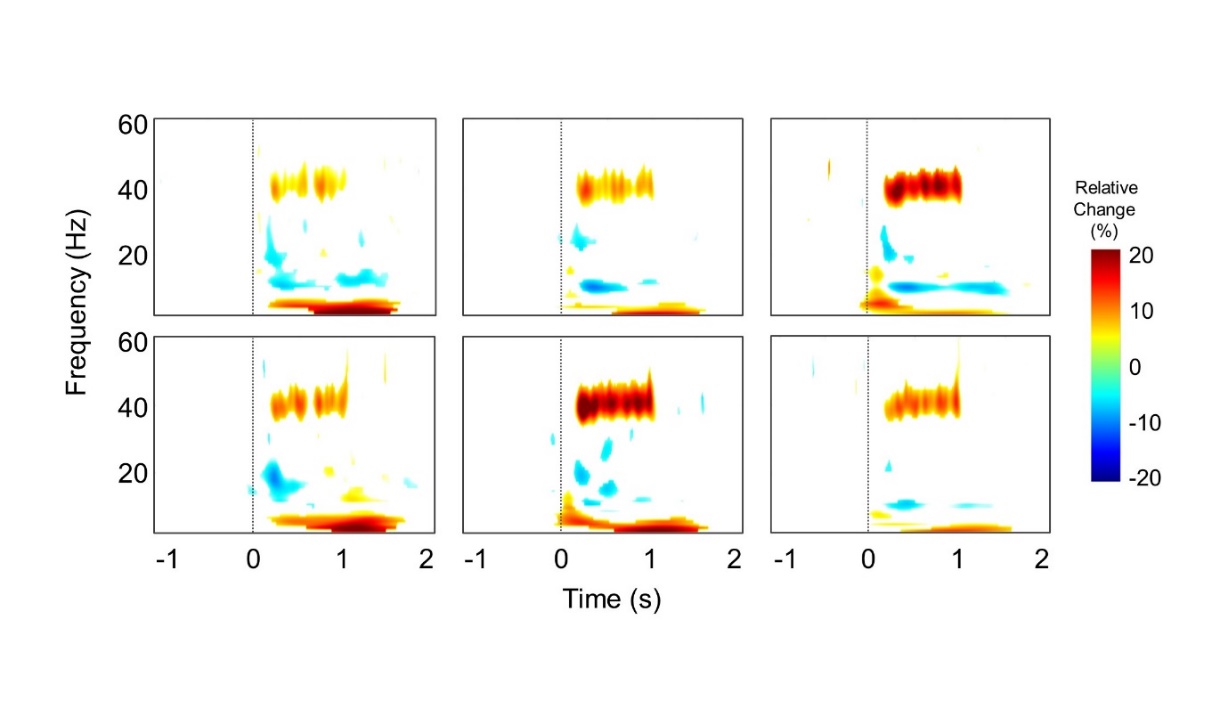
**

**Supplementary Figure 4. Grand-average time–frequency maps of statistical t-values for each OPM sensor.** The maps show the power changes of statistically significant differences relative to the baseline period (−1.1 to −0.2 s) obtained in a parametric t-test with a correction of the false discovery rate for multiple comparisons.
